# *Trichosanthes kirilowii* Extract Promotes Wound Healing through the Phosphorylation of ERK1/2 in Keratinocytes

**DOI:** 10.3390/biomimetics7040154

**Published:** 2022-10-07

**Authors:** Minho Kim, Jae-Goo Kim, Ki-Young Kim

**Affiliations:** 1 Graduate School of Biotechnology, Kyung Hee University, Yongin-si 446-701, Gyeonggi-do, Korea; 2 Department of Genetics and Biotechnology, College of Life Science, and Graduate School of Biotechnology, Kyung Hee University, Yongin-si 446-701, Gyeonggi-do, Korea

**Keywords:** HaCaT cell, MAP kinase pathway, wound healing, angiogenesis, AP-1 transcription factor, growth factor

## Abstract

The proliferation of keratinocytes is one of the important steps in the wound-healing process, which is regulated by various signals. Prior studies have shown that *Trichosanthes kirilowii* extract has the ability to promote angiogenesis. Therefore, in this study, we tested the wound-healing efficacy of *Trichosanthes kirilowii* extract with respect to promoting keratinocyte proliferation. A total of 100 μg/mL of *Trichosanthes kirilowii* extract treatment improved 145.38% of keratinocyte proliferation compared with DMSO-treated control in an MTT assay and increased 238.2% of wound closure by re-epithelialization in an in vitro wound-healing assay. *Trichosanthes kirilowii* extract promoted ERK1/2 phosphorylation in western blot analysis and induced the expression of the c-fos and c-jun (AP-1 transcription factors), cyclins (cell cycle regulator), and growth factors CTGF and VEGF (stimulator of angiogenesis) in qRT-PCR analysis. An in vivo wound-healing assay showed that *Trichosanthes kirilowii* extract improved wound healing, and the significant difference in wound closure compared with DMSO-treated control was shown on days 6 and 7 with a mouse model. Taken together, we demonstrate that *Trichosanthes kirilowii* extract promotes the proliferation of keratinocytes by activating ERK1/2 and increasing the mRNA expression of c-fos, c-jun, CTGF, and VEGF. Therefore, we suggest *Trichosanthes kirilowii* extract as a new component for skin care and as a wound-healing substance.

## 1. Introduction

Human skin constitutes the outermost part of the body and is important for maintaining homeostasis. The outermost structure of the skin is the stratum corneum, which is formed by flat hexagonal-shaped keratinocytes and is constantly regenerated by cell division in the lower layer [1]. After wound generation in the skin, rapid repair is required to protect the body. Wound-healing stages include the coagulation and hemostasis phase, inflammatory phase, proliferative phase, and remodeling phase [2]. In the wound-healing process, the roles of keratinocytes are filling the wound site through proliferation and migration and forming the outer skin layer through maturation and differentiation [3]. Thus, the promotion of keratinocyte proliferation is an important step in stimulating the skin wound-healing process.

The cell proliferation is regulated by various intracellular signaling pathways, including the mitogen-activated protein (MAP) kinase and phosphoinositide 3 kinase (PI3K)/protein kinase B (AKT) signaling pathway [4]. The MAP kinase signaling pathway is classified into three families: extracellular signal-regulated kinases (ERK), c-Jun N-terminal kinases (JNK), and p38 mitogen-activated protein kinases (p38) [5].

ERK1/2 is activated by MEK in accordance with the cell growth signal, and activated ERK1/2 phosphorylates nuclear targets such as Elk1. Elk1 regulates the expression of c-fos, and, finally, c-fos promotes cyclin D transcription [6,7]. Moreover, the activation of ERK1/2 is important for cell cycle progression in epidermal skin cell lines (keratinocytes and fibroblasts) [8,9]. The PI3K/AKT signaling pathway is also activated by extracellular growth signals such as growth factors, cytokines, and hormones. Activated AKT phosphorylates the mammalian target of rapamycin complex 1 and ribosomal protein S6 kinase to promote proliferation [4,10].

Activating protein-1 (AP-1) transcription factors regulate the expression of genes for differentiation, apoptosis, and proliferation-regulating proteins [11]. Especially in the proliferating cell, the expression of the AP-1 transcription factors c-jun and c-fos is highly increased and acts as a major player in cell cycle progression. The expression of c-fos is increased by the stimulation of TGFα, TGFβ, and growth factors [12,13].

*Trichosanthes kirilowii* (TK, Korean name is Haneultari) is a perennial vine plant belonging to the gourd family and is widely distributed in Northeast Asia, including Korea, China, Japan, and Mongolia. Traditionally, TK extract has been used as an expectorant and an antitussive and as a medicine for burns and frostbite [14]. Recent research showed that TK extract has anticancer, antibacterial, and anti-inflammatory properties [15,16,17,18]. TK extract has shown wound-healing potential in the CAM (Chick Chorioallantoic Membrane) assay through the promotion of angiogenesis [19].

In this study, our purpose was to verify the ability of TK extract to promote proliferation on keratinocytes and to confirm that TK extract has in vitro and in vivo wound-healing activity through ERK1/2 activation.

## 2. Materials and Methods

### 2.1. Plant Material and Extraction

Trichosanthes kirilowii was obtained from Jeju Island in South Korea. The whole part of the plant was dried with a freeze dryer (IlShin, Dongducheon, Korea). A total of 500 g of dried TK was extracted with 7.5 L of distilled water at 60 °C for 3 h. The extract was concentrated under reduced pressure at 50 °C using a rotary evaporator (EYELA, Tokyo, Japan) connected to a refrigerated bath circulator (JEIO TECH, Daejeon, Korea). The extract was filtered using muslin cloth followed by Whatman (GE Healthcare, Chicago, IL, USA) grade 1 filter paper and lyophilized using a freeze dryer. The extract was dissolved in dimethyl sulfoxide (DMSO) (10 mg/mL) and stored at 4 °C [20,21].

### 2.2. Cell Culture Conditions and TK Extract Treatment

The human keratinocyte cell line HaCaT [22] and human foreskin fibroblast cell line Hs68 (ATCC*^®^* CRL-1635™) were cultured in Dulbecco’s Modified Eagle’s Medium (10% Fetal Bovine Serum and 1% penicillin-streptomycin) at 37 °C in a 5% CO_2_ atmosphere. HaCaT and HS68 cells were seeded in 96-well plates (10^3^ cells per well) or 6-well plates (3 × 10^5^ cells per well). Then, 24 h after seeding, the medium was replaced with a serum-free medium containing indicated concentrations of TK extract, and the cells were incubated for an additional 24 h [22].

### 2.3. MTT Assay

HaCaT and HS68 cells were seeded in 96-well plates (10^3^ cells per well). After 24 h of seeding, the medium was replaced with a serum-free medium containing various concentrations of TK extract (0, 1, 5, 10, 50, and 100 μg/mL) and cultured for 24 h. MTT (3-(4,5-dimethyl-thiazol-2-yl)-2,5-diphenyltetrazolium bromide (Sigma Aldrich, St. Louis, MO, USA) was treated as 0.5 mg/mL. DMSO was used to solubilize formazan. The viability of cells was calculated from optical density (OD_540_) values measured using a microplate reader (BioTek Instruments, Winwooski, VT, USA). The compounds used in the MTT assay, including syringic acid (S6881), hesperetin (H4125), and furfural (185914), were purchased from Sigma Aldrich (St. Louis, MO, USA), and vanillic acid (CFN99331), diosmetin (CFN90210), and quercetin (CFN99272) were purchased from ChemFaces (Wuhan, China). The compounds were dissolved in DMSO (10 mg/mL) and stored at −20 °C. The experiments were independently repeated three times [22,23].

### 2.4. In Vitro Wound Healing Assay

HaCaT cells (3 × 10^5^ cells per well) were seeded into 6-well plates and cultured to form a monolayer (90–100% confluence). Using a 0.1–10 uL white pipet tip (Sorenson, Salt Lake City, UT, USA), a parallel linear wound was generated. The medium was replaced with a serum-free medium containing TK extract (0, 1, 10, and 100 μg/mL). The wound closure rate was calculated by comparing the wound area immediately after the scratch (0 h) and at 24 h. The data were analyzed by taking 10 consecutive pictures of 1 mm in length from the center of the well. The pictures of the wound area were taken using the EVOS XL imaging system (Fisher Scientific, Hampton, NH, USA), and the wound area was measured with the Image J program. The wound closure fold change was calculated by comparing the wound closure area (µm^2^) of each sample relative to the control. The experiments were independently repeated three times [22].

Wound closure area (µm^2^) = 0 h wound area − 24 h wound area.

### 2.5. Western Blot

HaCaT cells (3×10^5^ cells per well) were seeded into 6-well plates and incubated for 24 h. The medium was replaced with a serum-free medium containing indicated concentrations of TK extract (0, 1, 10, and 100 μg/mL), and the cells were incubated for an additional 24 h. A total of 20 μg of whole-cell lysate proteins were loaded in each lane of 6–10% of acrylamide SDS-PAGE gel. Antibodies for p38 (sc-535), JNK1/2 (sc-7345), p-JNK1/2 (sc-6254), ERK5 (sc-398015), p-ERK5 (sc-135760), GAPDH (sc-25778), and goat anti-rabbit IgG-HRP secondary antibody were purchased from Santa Cruz Biotechnology (Dallas, TX, USA). Antibodies for p-p38 (#9211), ERK1/2 (#9102), p-ERK1/2 (#9101), AKT (#9272), and p-AKT (9271) were purchased form Cell Signaling Technology (Danvers, MA, USA), and goat anti-mouse IgG-HRP secondary antibody was purchased from Bio-Rad (Hercules, CA, USA). Polyvinylidene fluoride (Bio-Rad, Hercules, CA, USA) membrane and bovine serum albumin (Roche, Basel, Switzerland) were used. The membranes were developed using enhanced ECL (Bio-Rad, Hercules, CA, USA) on a UVITEC imaging system (UVITEC, Cambridge, UK) [22].

### 2.6. Quantitative Real-Time PCR

HaCaT cells (3 × 10^5^ cells per well) were seeded into 6-well plates and incubated for 24 h. The medium was replaced with a serum-free medium containing indicated concentrations of TK extract (0, 1, 10, and 100 μg/mL), and the cells were incubated for an additional 24 h. The TRIzol reagent (Fisher Scientific, Hampton, NH, USA) was used in the total RNA extraction, and Thermo reverse transcriptase (NanoHelix, Daejeon, Korea) was used for cDNA synthesis (1 μg of total RNA). The total RNA extraction and cDNA synthesis followed the manufacturer’s manual. The cDNA was used as the template for real-time PCR, which was carried out with QGreen 2× SybrGreen qPCR Master Mix (CellSafe, Yongin, Korea). The primer sequences are listed in Table 1. GAPDH was used as a quantitative control. Gene expression fold change was calculated using the delta-delta Cq formula. The experiments were independently repeated three times [21].

### 2.7. In Vivo Wound-Healing Assay

Female (BALB/cAnNTac) 6~7-week-old mice were purchased from JA BIO (Suwon, Korea). The mice were anesthetized by the intraperitoneal injection of 125 mg/kg of Avertin (2,2,2-Tribromoethanol, Sigma Aldrich, St. Louis, MO, USA). The groups were randomly selected, and one mouse was housed per cage (12 h day–night cycle, temperature: 20–26 °C, humidity: 45–55%). An adaptation period was given for 7 days. The hair on the back of the mouse was cut with an animal clipper (JEUNG DO BIO & PLANT CO, Seoul, Korea), and a Hair Removal Cream (BEAUTY FORMULAS, Leeds, UK) was applied to completely remove the hair. After 3 days, a 4 mm-diameter Keyes biopsy punch (Olynth Surgical, Sialkot, Pakistan) was used to generate the full thickness of the back skin below the shoulder blades. Two wounds were formed per individual mouse; the left wound was used as a control (DMSO) and the right wound was treated with TK extract (50 μg/day). The wound healing process was photographed daily. The wound closure (%) was calculated using the formula below. Three animals were used as one experimental group. The mice were euthanized by cervical dislocation after anesthesia. This study was conducted with the approval of the Animal Experimental Ethics Committee of Kyung Hee University (approval number: KHGASP-20-560) and performed under the Institutional Animal Care and Use Committee (IACUC) guidelines. The experiments were independently repeated three times [31,32].

(1) $$\mathrm{Wound}\;\mathrm{closure}\;\left(\%\right)=\frac{D0\;\mathrm{wound}\;\mathrm{area}-Dx\;\mathrm{wound}\;\mathrm{area}}{D0\;\mathrm{wound}\;\mathrm{area}}\times 100$$

### 2.8. Statistical Analysis

The results are expressed as the means ± standard deviation. Statistically significant differences were analyzed using a one-way ANOVA. Student’s t-test was used when only two groups were compared (*: *p* < 0.05, **: *p* < 0.01, ***: *p* < 0.001).

## 3. Results

### 3.1. TK Extract Induced the Proliferation of the Keratinocyte Cell

**Figure 1:**
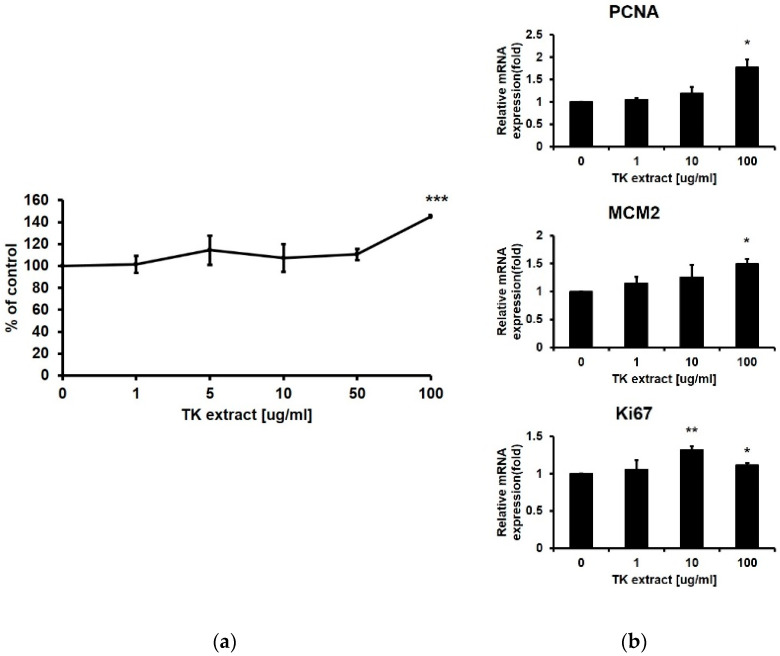
*Trichosanthes kirilowii* (TK) extract induced the proliferation of HaCaT cells. (**a**) HaCaT cells were treated with indicated concentrations of TK extract. The cell proliferation rate was evaluated based on the MTT assay. (***: *p* < 0.001.) (**b**) TK extract dose-dependently increased the mRNA expression of proliferation marker genes in HaCaT cells based on the qRT-PCR analysis. (*: *p* < 0.05, **: *p* < 0.01.)

An MTT assay was performed using HaCaT cells to check the proliferative effect of TK extract (0, 1, 5, 10, 50, and 100 μg/mL). TK extract dose-dependently increased the proliferation rate by 145.38% at 100 μg/mL of the TK extract treatment compared to the control (DMSO) (Figure 1a). At concentrations above 100 μg/mL, the MTT assay value decreased.

qRT-PCR was performed with proliferation markers including Ki67, MCM2, and PCNA [33]. The mRNA expression levels of all three proliferation markers were dose-dependently increased by the treatment of TK extract (Figure 1b). These results indicated that TK extract promotes keratinocyte proliferation.

### 3.2. TK Extract Promoted In Vitro Wound-Healing Activity

**Figure 2:**
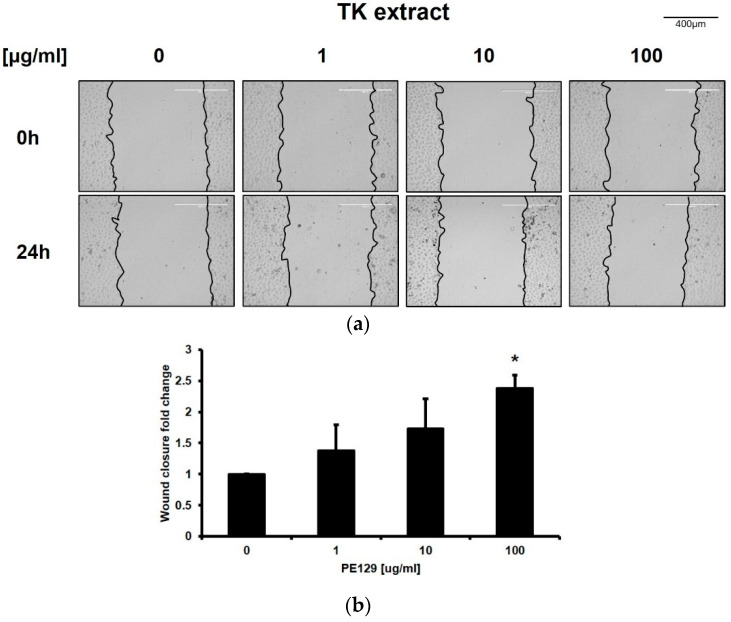
TK extract promoted the in vitro wound healing of HaCaT cells. HaCaT cells were cultured in a 6-well plate, scratched, and treated with the indicated concentration of TK extract. After 24 h, the scratched area was measured and compared. (**a**) The results were imaged to show scratched wound healing. (**b**) The fold change graph of (**a**). (*: *p* < 0.05.)

The wound-healing assay was performed in in vitro conditions to verify the improvement of keratinocyte re-epithelialization by TK extract treatment. An artificial wound was created on the monolayer of HaCaT cells, and wound closure was observed 24 h after TK extract treatment. The TK extract treatment increased wound closure by 138.2% (1 μg/mL), 173.4% (10 μg/mL), and 238.2% (100 μg/mL), respectively, compared to the non-treated control (DMSO) (Figure 2). This result indicated that TK extract promotes the re-epithelization of keratinocytes by activating the proliferation.

### 3.3. TK Extract Induced ERK1/2 Phosphorylation

**Figure 3:**
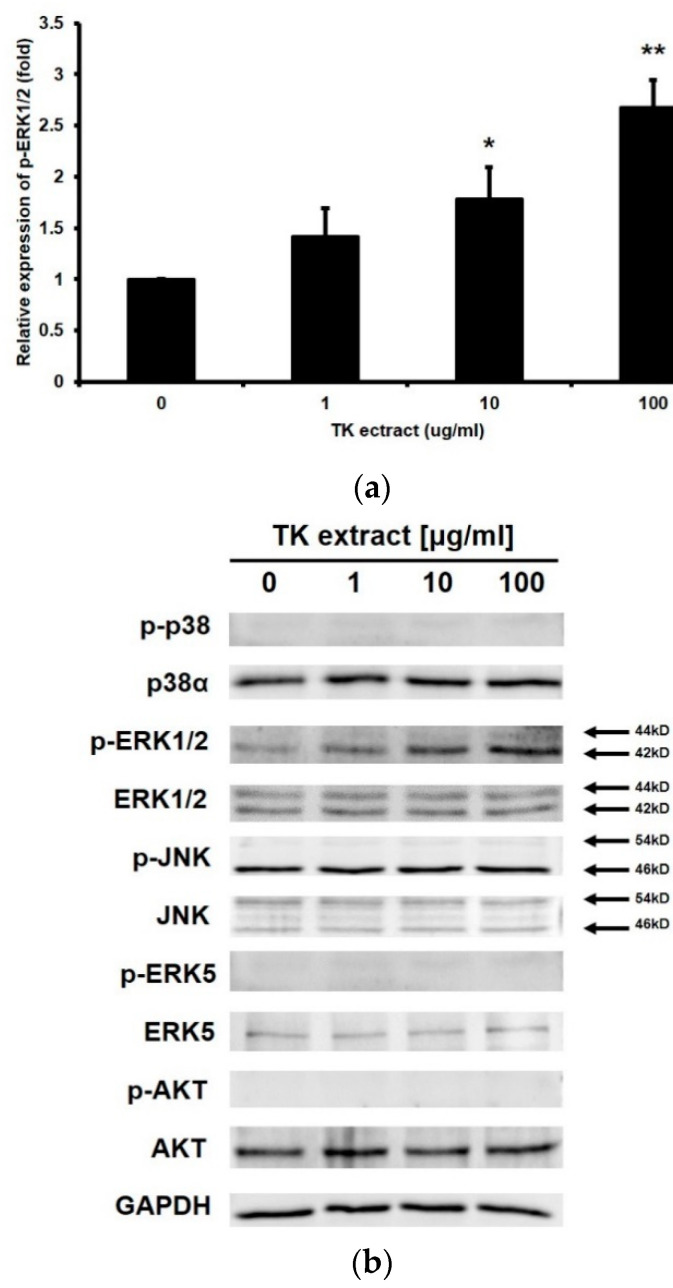
TK extract induced ERK1/2 phosphorylation in HaCaT cells. HaCaT cells were treated with the indicated concentration of TK extract, and the whole cell lysate proteins were used in western blot analysis. (**a**) GAPDH was used as a quantitative control. Phosphorylated forms of p38, ERK1/2, JNK, ERK5, and AKT were detected. (**b**) The p-ERK1/2 band was quantified with densitometric analysis and normalized to GAPDH. (*: *p* < 0.05, **: *p* < 0.01.)

The MAP kinase pathway and the PI3K/AKT pathway are involved in keratinocyte proliferation and migration. Western blotting was performed to check the expression and the phosphorylation of these MAP kinases (p38α, ERK1/2, JNK, and ERK5) and AKT. The amount of the phosphorylated form of ERK1/2 increased depending on the concentration of the treated TK extract (Figure 3). Except for ERK1/2, MAP kinases or AKT were not activated. These results suggested that TK extract promotes keratinocyte proliferation through the ERK1/2 pathway.

### 3.4. TK Extract Induced the mRNA Expression of Proliferation Regulatory Genes

**Figure 4:**
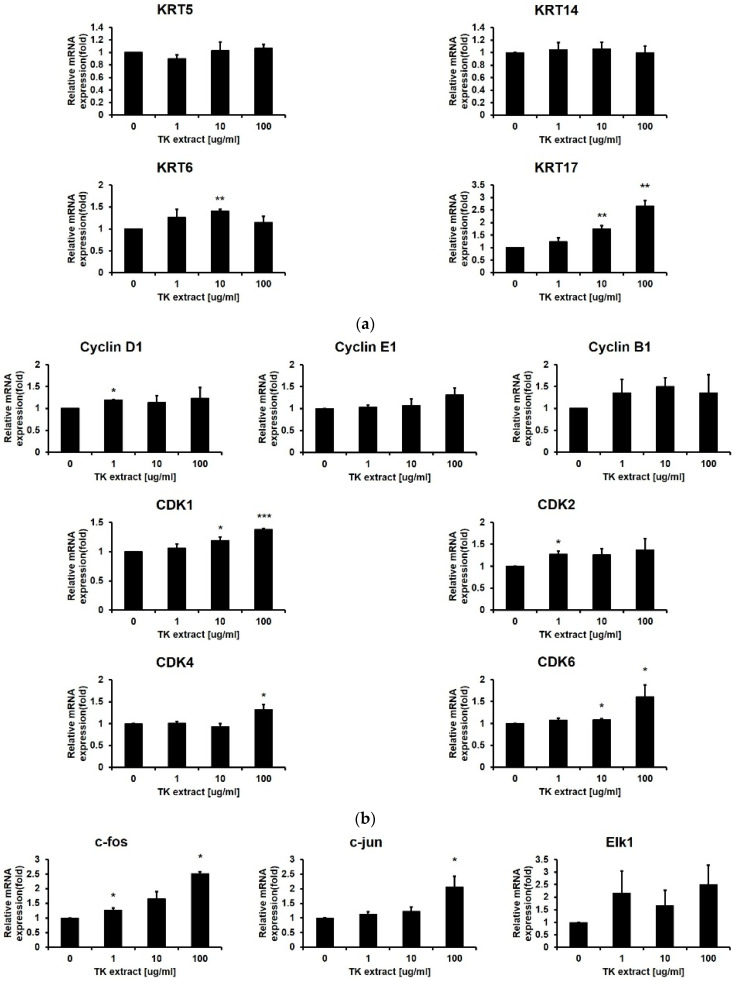

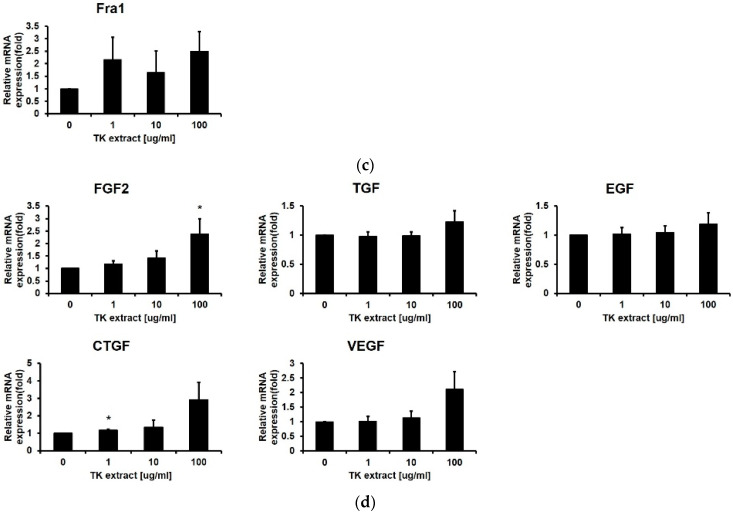
TK extract induced the mRNA expression of cell proliferation regulatory genes in HaCaT cells. HaCaT cells were treated with the indicated concentration of TK extract, and the total mRNA was used for qRT-PCR. The mRNA expression of (**a**) keratin genes (KRT), (**b**) cyclins and CDKs, (**c**) AP-1 transcription factors and Elk1, and (**d**) growth factors was analyzed. (*: *p* < 0.05, **: *p* < 0.01, ***: *p* < 0.001.)

KRT5/14/6/17 genes are keratin protein-coding genes and are specifically expressed in keratinocytes. These KRT genes are one of the proliferation markers in keratinocytes. Even though TK extract did not induce the mRNA expression of KRT5/14, which expresses in the basal proliferative keratinocytes in the quiescent epidermis, TK extract induced the mRNA expression of KRT17, which is highly expressed in the hyper-proliferative cellular responses condition (KRT17: 265% at 100 μg/mL) (Figure 4a) [34].

After the ERK1/2 pathway is identified as a target for the TK extract to activate keratinocyte proliferation, qRT-PCR was performed for genes related to cell proliferation regulated by ERK1/2. Cyclins and CDKs are the main regulators for cell cycle progression. The mRNA expression of Cyclin D1/B1/E1 and CDK1/2/4/6 has a positive correlation with the concentration of TK extract treatment (Figure 4b). The AP-1 transcription factor (c-fos, c-jun, and Fra-1) and Elk1 regulate the transcription of cell cycle-related genes, and their mRNA expression showed a positive correlation with TK extract treatment. The c-fos and c-jun mRNA expression was especially dose-dependently increased by the treatment of TK extract (c-fos: 251.5% at 100 μg/mL, and c-jun: 206.4% at 100 μg/mL, respectively) (Figure 4c).

Growth factors stimulate cell growth by activating the MAP kinase pathway. The mRNA expression of growth factor genes also showed a positive correlation with TK extract treatment. Fibroblast growth factor 2 (FGF2) increased by 237.7% at 100 μg/mL, vascular endothelial growth factor (VEGF) was increased by 211.7% at 100 μg/mL, and connective tissue growth factor (CTGF) increased by 291.9% at 100 μg/mL of TK extract treatment (Figure 4d). These results indicated that the treatment of TK extract activates the hyper-proliferative signal, and c-fos, c-jun, FGF2, VEGF, and CTGF are highly related to proliferation activated by the TK extract.

### 3.5. TK Extract Influenced Fibroblast mRNA Expression

**Figure 5:**
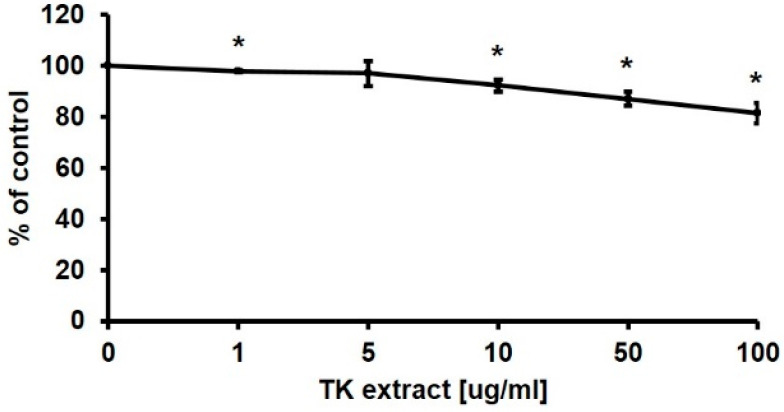
MTT assay results of Hs68 cells after TK extract treatment. Hs68 cells were treated with indicated concentrations of TK extract. The cell viability was evaluated with an MTT assay. (*: *p* < 0.05.)

In the wound-healing process, fibroblasts are also important in creating a new extracellular matrix and collagen structure. In contrast to keratinocytes, fibroblasts did not show a significant increase in proliferation by the treatment of TK extract in the MTT assay (Figure 5).

**Figure 6:**
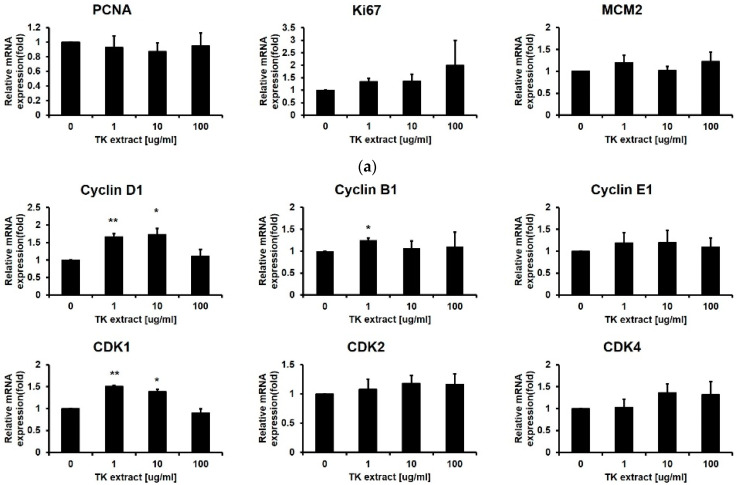

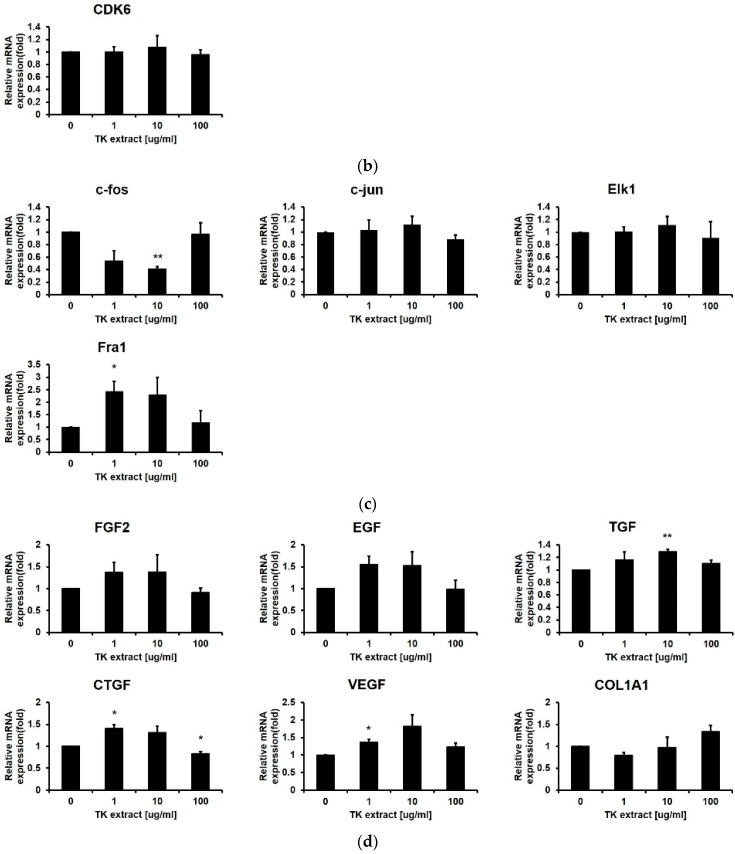
Hs68 cells’ mRNA expression changes after TK extract treatment. Hs68 cells were treated with the indicated concentration of TK extract, and the total mRNA was used in qRT-PCR. The mRNA expression of (**a**) proliferation marker genes, (**b**) cyclins and CDKs, (**c**) AP-1 transcription factors and Elk1, and (**d**) growth factors and collagen gene (COL1A1) was analyzed. (*: *p* < 0.05, **: *p* < 0.01.)

The mRNA expression of genes related to cell proliferation regulation in fibroblasts was also tested by qRT-PCR. The mRNA expression change of the Hs68 cells was not significant, except for Cyclin D1 (172.9% at 10 μg/mL), CDK1 (150.8% at 1 μg/mL), EGF (155.4% at 1 μg/mL), and VEGF (181.9% at 10 μg/mL) (Figure 6). Taken together, in fibroblasts, proliferation was not promoted as in keratinocytes, and significant changes in gene expression were not observed. The efficacy of TK extract on fibroblasts appears to be minor.

### 3.6. TK Extract Promoted In Vivo Wound-Healing Activity

**Figure 7:**
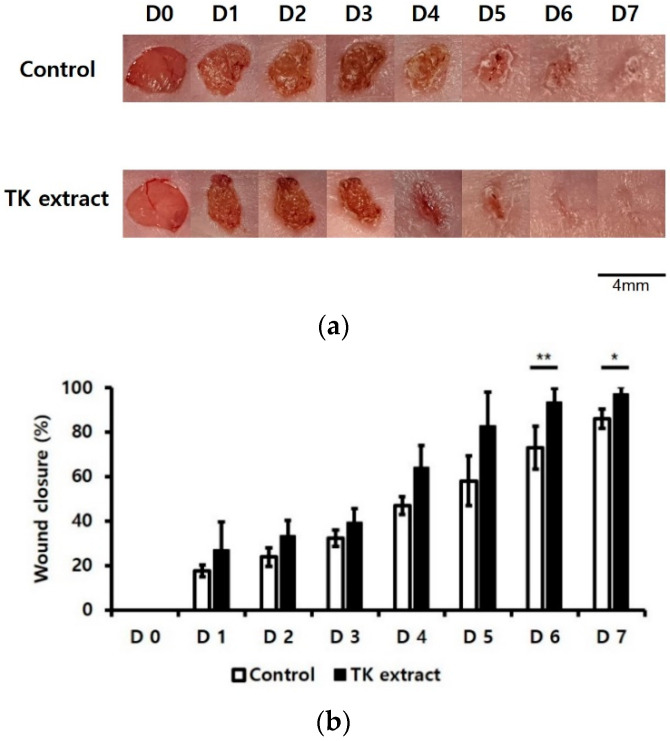
TK extract promoted in vivo wound healing. In vivo wound-healing activity was measured using BALB/c mice. The 4 mm-diameter wounds were created by both backsides of 6~7-week-old BALB/c mice. (**a**) The results were imaged to show wound healing. (**b**) The wound closure (%) graph of (**a**). (*: *p* < 0.05, **: *p* < 0.01.)

An in vivo wound healing assay was performed to verify the wound-healing effect of the TK extract. Wounds were formed on both sides of the back skin. The treatment of the TK extract improved the wound closure compared to the control wound area (Figure 7). The efficacy of wound healing started to be seen from the 5th day, and there was a significant difference in the wound closure of the control and the TK extract-treated sample on the 6th and 7th days.

### 3.7. Effect of Constituent Compounds of TK Extract on the Proliferation of HaCaT Cells

**Figure 8:**
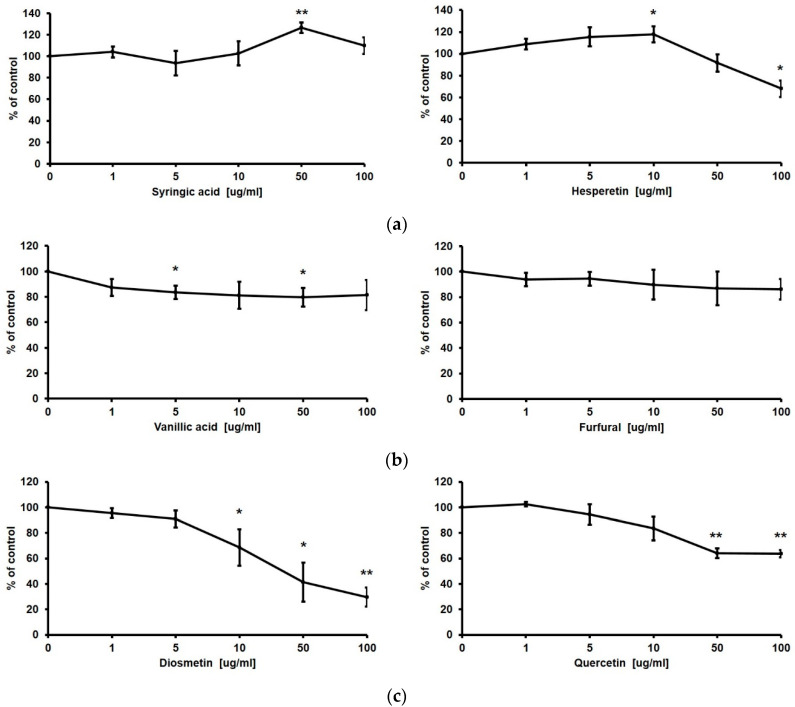
Proliferative effect of constituent compounds of TK extract on HaCaT cells. HaCaT cells were treated with indicated concentrations of syringic acid and hesperetin (**a**), vanillic acid and furfural (**b**), and diosmetin and quercetin (**c**). The cell viability was evaluated with an MTT assay. (*: *p* < 0.05, **: *p* < 0.01.)

Finally, we checked the efficacy of the constituent compounds of TK extract on the proliferation of HaCaT cells. Gentisic acid, syringic acid, hesperetin, vanillic acid, furfural, diosmetin, quercetin, chlorogenic acid, apigenin, caffeic acid, and gallic acid are known to be contained in *Trichosanthes kirilowii* [35,36,37]. Syringic acid and hesperetin promoted the proliferation of HaCaT cells; however, vanillic acid and furfural did not affect HaCaT cell proliferation, and diosmetin and quercetin rather reduced the proliferation of HaCaT cells based on the MTT assay results (Figure 8).

## 4. Discussion

Many studies have been conducted to find effective plant extracts for wound healing [38,39]. We tested TK extract as one of the plant-derived wound-healing substances. TK extract promoted the proliferation of keratinocytes (Figure 1), which is consistent with the previous study. TK extract induced a higher level of angiogenesis on bovine aortic endothelial cells than other plant extracts [19]. Angiogenesis is the process of promoting blood vessel formation, which promotes wound repair by forming new blood vessels in the wound area [40].

The progression of angiogenesis is promoted by various extracellular matrix materials, such as CTGF and VEGF [41]. Growth factors are one of the extracellular signaling materials for cell proliferation, wound healing, and differentiation [42]. CTGF and VEGF are growth factors that promote angiogenesis and are expressed in both keratinocytes and fibroblasts. The expression level of CTGF increases after wounding and is involved in granulation tissue formation, re-epithelialization, matrix formation, and remodeling [41,43]. VEGF also plays an important role in wound healing by inducing angiogenesis, the migration of endothelial cells, and vascular permeability [44,45].

TK extract induces the phosphorylation of ERK1/2 in keratinocytes (Figure 3). Previous studies have revealed a relationship between the phosphorylation of ERK1/2 and cell proliferation. However, ERK 1/2 is known to have about 200 substrates, and the phosphorylation of ERK 1/2 determines not only proliferation but also overall cell fate, including apoptosis the cell death [46]. It is unclear how the phosphorylation of ERK1/2 regulates the exact gene expression under various conditions. In the cell cycle progression of keratinocytes and fibroblasts, the sustained activation of ERK1/2 is essential, and it is important for both G1/S and G2/M transitions [8,47]. Activated ERK 1/2 inhibits the expression of cell cycle repressor genes, phosphorylates the nuclear targets, and regulates the expression of transcription factors such as AP-1 transcription factors and, finally, cyclins [6,14,47].

**Figure 9:**
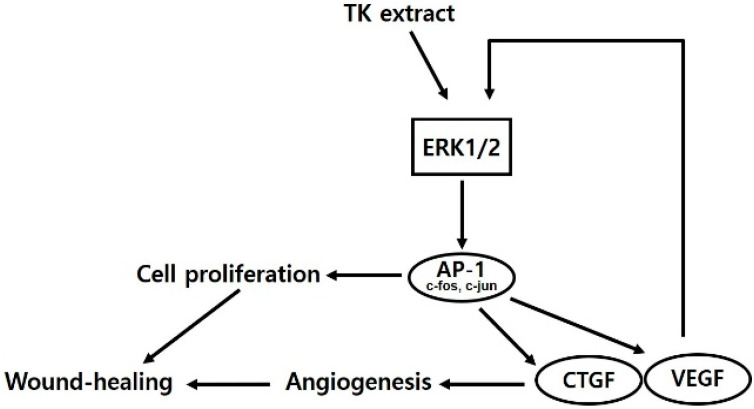
Schematic diagram of TK extract effects in keratinocytes.

TK extract dose-dependently promoted the mRNA expression of c-fos and c-jun and the AP-1 transcription factors, and the mRNA expression of cyclins and CDKs was increased (Figure 4). Expressed c-fos and c-jun induced cell proliferation by promoting the cell cycle regulatory gene expression and angiogenesis by inducing the expression of CTGF and VEGF [48,49,50]. TK extract did not substantially promote cell proliferation in fibroblasts (Figure 5), and it also did not show a significant increase in gene expression (Figure 6). Considered in connection with the in vivo wound-healing assay, the wound-healing activity of TK extract appears to be concentrated in keratinocytes (Figure 9).

TK extract consists of various components [35,36,37]. We checked the efficacy of several constituent compounds on the proliferation of HaCaT cells (Figure 8). Gentisic acid, syringic acid, hesperetin, and chlorogenic acid were already reported to promote cell proliferation and wound-healing activity [22,51,52,53], and diosmetin, quercetin, caffeic acid, apigenin, and gallic acid, which inhibited proliferation, were known to have anticancer efficacy according to previous studies [22,54,55,56]. In our previous study, we confirmed that gentisic acid promotes proliferation through the phosphorylation of ERK1/2 in keratinocytes [22]. Syringic acid increases the expression of TGF-β and collagen 1 in keratinocyte cells [51,57], hesperetin is known to increase the secretion of VEGF in keratinocytes [58], and chlorogenic acid increases the proliferation of fibroblasts and increases the expression of skin barrier genes such as collagen1 and KRT1 [59]. Taken together, the keratinocyte proliferation-promoting activity of TK extract appears to be a combination of four substances, and gentisic acid and hesperetin are thought to be key substances for ERK1/2- and VEGF-related mechanisms.

Our future study will focus on the efficacy of individual compounds included in the plant extract by identifying the intracellular target of the compound ((ex) ERK1/2) and checking the entire gene expression change pool after compound treatment. Finally, we will find the key genes that are important for the wound-healing process and investigate the mechanisms of transcription factors or the cofactors of those genes.

## 5. Conclusions

In this study, TK extract induced keratinocyte proliferation by ERK 1/2 phosphorylation and affected wound healing in both in vitro and in vivo conditions. TK extract could be considered as a lead component for wound healing and skin proliferation. Our further study will be focused on the mechanisms of keratinocyte proliferation after compound treatment compared with gentisic acid, which we reported before [22]. In order for it to be used as a constituent of pharmaceuticals, the discovery and study of key compounds are needed.

## Figures and Tables

**Table 1:** Primers used in this study.

Genes	Forward	Reverse	Reference
PCNA	AACCTCACCAGTATGTCCAA	ACTTTCTCCTGGTTTGGTG	[24]
KI67	CCAAAGAAGGCTGAGGACAA	CCCTTAAGCAGACTGACAGC	This study
MCM2	AATCTATGGCGACAGGCAG	ATCACATAGTCCCGCAGAT	This study
Cyclin D1	CTGTGCTGCGAAGTGGAAACC	GACGATCTTCCGCATGGAC	This study
Cyclin B1	TAAGGCGAAGATCAACATGG	GCTTCCTTCTTCATAGGCAT	This study
Cyclin E1	ACACCATGAAGGAGGACG	CACAGACTGCATTATTGTCCC	This study
CDK1	CAGGTCAAGTGGTAGCCATG	ACCTGGAATCCTGCATAAGC	This study
CDK2	TTCTCATCGGGTCCTCCACC	TCGGTACCACAGGGTCACCA	This study
CDK4	CTGAGAATGGCTACCTCTCG	CGAACTGTGCTGATGGGAAG	This study
CDK6	CCGAAGTCTTGCTCCAGTCC	GGGAGTCCAATCACGTCCAA	This study
KRT5	AGCAGTGGTACGCTTGTTGATT	GCCTGGACTCAGAGCTGAGAA	[25]
KRT14	GGCCTGCTGAGATCAAAGACTAC	CACTGTGGCTGTGAGAATCTTGTT	[26]
KRT6	CTGAGGCTGAGTCCTGGTAC	GTTCTTGGCATCCTTGAGG	This study
KRT17	GCTGCTACAGCTTTGGCTCT	TCACCTCCAGCTCAGTGTTG	[27]
c-fos	GGAGGAGGGAGCTGACTGATA	GCAATCTCGGTCTGCAA	[28]
c-jun	TTCTATGACGATGCCCTCAACGC	GCTCTGTTTCAGGATCTTGGGGTTAC	[28]
Fra-1	GGGCATGTTCCGAGACTTC	GCACCAGGTGGAACTTCTG	[29]
Elk1	CTGACCCCATCCCTGCTTCCTA	GAAGTGAATGCTAGGAGGCAGCG	[28]
FGF2	AAAAACGGGGGCTTCTTCCT	AGCCAGGTAACGGTTAGCAC	[30]
EGF	AGTCCGTGACTTGCAAGAGG	CCTCTTCTTCCCTAGCCCCT	[30]
TGF	TGGTGGAAACCCACAACGAA	GAGCAACACGGGTTCAGGTA	[30]
CTGF	GTTTGGCCCAGACCCAACTA	GGCTCTGCTTCTCTAGCCTG	[30]
VEGF	CTTGCCTTGCTGCTCTACCT	GCAGTAGCTGCGCTGATAGA	[30]
COL1A1	CATGACCGAGACGTGTGGAA	GGCAGTTCTTGGTCTCGTCA	[30]
GAPDH	GTATCGTGGAAGGACTCATG	GAGGCAGGGATGATGTTC	This study

## Data Availability

Not applicable.
